# Foodborne* Listeria monocytogenes*: A Real Challenge in Quality Control

**DOI:** 10.1155/2016/5768526

**Published:** 2016-04-30

**Authors:** Tünde Pusztahelyi, Judit Szabó, Zsuzsanna Dombrádi, Szilvia Kovács, István Pócsi

**Affiliations:** ^1^Central Laboratory, Faculty of Agricultural and Food Sciences and Environmental Management, University of Debrecen, Böszörményi Út 138, 4032 Debrecen, Hungary; ^2^Department of Medical Microbiology, Clinical Centre, University of Debrecen, Nagyerdei Körút 98, 4032 Debrecen, Hungary; ^3^Department of Biotechnology and Microbiology, Faculty of Science and Technology, University of Debrecen, Egyetem Tér 1, 4032 Debrecen, Hungary

## Abstract

*Listeria monocytogenes* is a foodborne pathogen, and the detection and differentiation of this bacterium from the nonpathogenic* Listeria* species are of great importance to the food industry. Differentiation of* Listeria* species is very difficult, even with the sophisticated MALDI-TOF MS technique because of the close genetic relationship of the species and the usual gene transfer. The present paper emphasizes the difficulties of the differentiation through the standardized detection and confirmation according to ISO 11290-1:1996 and basic available* L. monocytogenes* detection methods and tests (such as API* Listeria* test, MALDI-TOF MS analysis, and* hly* gene PCR). With the increase of reports on the pathogenesis of atypical* Listeria* strains in humans, the significance of species level determination has become questionable, especially in food quality control, and the detection of pathogenic characteristics seems to be more relevant.

## 1. Introduction

Out of the six closely related* Listeria* species (*L. monocytogenes*,* L. ivanovii*,* L. seeligeri*,* L. innocua*,* L. grayi*, and* L. welshimeri*) and the newly isolated nonpathogenic* L. marthii* [1],* L. rocourtiae* [2],* L. weihenstephanensis, *and* L. fleischmannii *[3],* L. monocytogenes* is generally regarded as the only one that is capable of producing human listeriosis [4] with a high mortality rate (greater than 20%). Therefore, laboratory diagnosis has focused primarily on the detection of* L. monocytogenes* and its differentiation from other* Listeria* species [5]. In food samples, the contamination level is usually low (10 CFU/g), but higher than 100 CFU/g can be detected in some cases, for example, in minced meat or smoked fish [6]. Previously, some practical approaches to differentiation tests included culturing with and without preenrichment or with direct analysis from the food sample with biochemical confirmation tests and/or nucleic acid based techniques (reviewed by Churchill et al. [7]), and these methods presented some success for the food industry. Others approached* Listeria* differentiation from a technical point of view by applying money-consuming methods for precise strain differentiation which is especially good for medical and/or research purposes (reviewed by Gasanov et al. [8]).

The standardized* L. monocytogenes* detection method in Europe requires a laborious testing scheme which is time-consuming, taking five to six days [9, 10]. Because of the high genome homology among* Listeria* species [11–13] the strains display similar morphological, biochemical, serological, and molecular characteristics [4, 14] that causes difficulty of the identification.

In* L. monocytogenes*, the central virulence gene cluster (pathogenicity island, e.g., LIPI-1) codes for the main activities that have importance in the intracellular life of the microbe inside the host. According to some researchers, nonpathogenic* L. innocua* species have lost their pathogenicity island [4, 11]. However, Johnson et al. [15] detected* hly* and* mpl* genes of the LIPI-1 pathogenicity island in atypical* L. innocua* and there are also reports on human infections caused by* L. innocua* [16],* L. seeligeri *[17], and* L. ivanovii* [18]. Here, a question is arising: what is more important in food hygiene? The species level determination of* L. monocytogenes* or the enumeration of possibly virulent* Listeria* species?

Here, ALOA medium, API* Listeria* biochemical test strip, MALDI-TOF MS analysis, and* hly* gene detection by PCR were compared to the standardized detection and confirmation according to ISO 11290-1:1996 on foodborne and reference strains.

## 2. Materials and Methods

### 2.1. Reference Strains

The following type strains were purchased from the National Collection of Agricultural and Industrial Microorganisms (NCAIM), Faculty of Food Sciences, Corvinus University of Budapest (Hungary):* Listeria monocytogenes* NCAIM B 01934 (ATCC 15313) type strain;* Listeria monocytogenes* NCAIM B 01966 (ATCC 35152) nonhaemolytic nonvirulent variant [19];* L. innocua* NCAIM B01933 (ATCC 33090) serotype 6a;* L. welshimeri* NCAIM B01872 (ATCC 35897) serotype 6b;* L. seeligeri* NCAIM B01873 (ATCC 35967);* L. ivanovii* NCAIM B02035 (ATCC 19119);* L. grayi* ssp*. murray* NCAIM B01372 (ATCC 25401);* L. grayi* ssp*. grayi* NCAIM B01871 (ATCC 19120).

### 2.2. Isolation of Foodborne* Listeria*

All applied enrichment media, selective media, and supplements were identical in composition to the described media of the ISO 11290-1:1996 standard [9] and supplied in dehydrated form. All media were tested with the* L. monocytogenes* NCAIM B 01934 reference strain.

Thirty-one commercially available frankfurter products originated from different producers were sampled and analyzed. From each of them 25 g was measured into 225 mL preenrichment medium (half Fraser broth, Merck, Hungary) in sterile bags and was homogenized in a Stomacher homogenizer (IUL Instruments, Spain) for 1 minute [9].

The homogenized samples were incubated in Schott flasks with loosening caps for 24 hours at 30°C. From each culture 0.1 mL was inoculated into 10 mL enrichment Fraser broth and incubated at 37°C for 48 hours. Black color development, which is characteristic of* Listeria* species, was detected in some of the culture broths. All cultures were streaked onto Oxford and PALCAM selective agar plates (Merck, Hungary) and incubated at 37°C for 24–48 hours. All colonies showed homogeneous morphology on these media. Subcultures of colonies were tested on sheep blood agar plates (OXOID, UK) (24 ± 2 hours) and with the Christie-Atkins-Munch-Petersen (CAMP) test for enhanced beta-haemolysis; on ALOA plates (VWR BDH Prolabo, USA) for *β*-glucosidase and phospholipase activities, catalase test and Gram staining were also performed. Gram-positive and catalase positive bacterial strains were stored in a Cryobank system (Mast Diagnostics, UK) at −75°C and were applied for further testing.

### 2.3. Biochemical Confirmation Tests

The biochemical confirmation reactions were performed with the API* Listeria* test (bioMérieux, France) according to the instructions of the manufacturer using bacterial cultures cultivated freshly on sheep blood agar plates (OXOID, UK). Reading of the API test strips was evaluated with the mini-API instrument (bioMérieux, France).

### 2.4. MALDI-TOF MS

Bacterial isolates and* Listeria* reference strains were grown overnight at 37°C on sheep blood agar (OXOID, UK). Cells of a whole colony were placed in a spot on the steel target plate. Each sample was overlaid with 1 *μ*L of matrix solution (saturated solution of *α*-cyano-4-hydroxycinnamic acid in 50% acetonitrile, 2.5% trifluoroacetic acid) and air-dried at room temperature. MALDI-TOF MS was performed on a Microflex LT instrument (Bruker Daltonik GmbH, Germany). For automated data analysis, raw spectra were processed using the MALDI Biotyper 3.0 software (Bruker Daltonik GmbH, Germany) with default settings. Generated peak lists were used for matches against reference libraries directly using the integrated pattern matching algorithm of the MALDI Biotyper 3.0 software [20].

### 2.5. Molecular Biological Identification

Genomic DNA was isolated from cultures of 1.5 mL of brain heart infusion broth (LAB M Limited, UK) or Tryptic Soy Broth (OXOID, UK) containing inoculated frankfurter products, using the Wizard Genomic DNA Purification Kit (Promega, USA) according to the instructions of the manufacturer. The integrity of the genomic DNA was tested by analyzing the samples on 0.8% agarose gel stained with ethidium bromide. Purity and concentration of the DNA were determined spectrophotometrically. Primers were designed with the Oligo Explorer software (version 1.2; Gene Link, http://www.genelink.com/), evaluated with the OligoAnalyzer (version 1.1.2., Teemu Kuulasma) software, and purchased from Integrated DNA Technologies (USA). Oligonucleotides used in the PCR reactions were the* hlyQF/hlyQR* primers of Rodríguez-Lázaro et al. [21] and the following designed primer pair:

 hly F: 5′-CACTCAGCATTGATTTGCCA-3′,
 hly R: 5′-CATACGCCACACTTGAGATA-3′.

PCR reactions for the* hlyF/hlyR* primers were run under the following conditions: the final volume of the 50 *μ*L PCR solution contained 2–4 *μ*L of genomic DNA, 5 *μ*L of 10x Taq buffer, 5 *μ*L 25 mM of MgCl_2_, 2-2 *μ*L of each 10 *μ*M primer, 4 *μ*L of 10 mM dNTP mixture, and 2.5 U Taq polymerase (Fermentas, Germany). In the case of the* hlyQF/hlyQR* primers, PCR reaction conditions were determined by Rodríguez-Lázaro et al. [21]. The annealing temperature in the case of our* hlyF/hlyR* primers was 55°C. The amplification conditions were 3 min denaturation at 96°C, 35 cycles each consisting of 30 s at 94°C, 45 s at the corresponding annealing temperature, and 1.3 min at 72°C followed by a final extension for 10 min at 72°C. We applied* L. monocytogenes* NCAIM B 01934 genomic DNA template as a positive control in each PCR run.

Validation of the PCR protocol was carried out with artificially contaminated food samples. 25 g portions of commercial pasteurized frankfurter were suspended in 225 mL TSB (Tryptic Soy Broth; OXOID, UK) and homogenized in a Stomacher homogenizer (IUL Instruments, Spain) for 1 minute. The suspensions were inoculated with decimal dilutions of exponential phase culture of the* L. monocytogenes* NCAIM B 01934 reference strain suspension from 1 to 10^5^ cells/mL concentrations, mixed well, and samples were taken immediately after inoculation. Following DNA isolation,* hlyF/hlyR* primers were applied in the PCR reactions. PCR products were sequenced in order to test their identity (data not shown). Reference sequences were gained from the National Center for Biotechnology Information Database (http://www.ncbi.nlm.nih.gov/) and aligned with the sequenced PCR products using the BioEdit Biological Sequence Alignment Editor Program [22].

## 3. Results and Discussion

From 31 frankfurter samples, 11* Listeria* strains were isolated with the standardized method of* L. monocytogenes* detection. The isolates produced typical colonies on PALCAM agar (except Isolate 2 on Table 1) and/or Oxford selective agar media were Gram-positive and catalase positive.

*L. monocytogenes* should show haemolysis on sheep blood agar plates or with* Staphylococcus aureus* with the CAMP test and utilize rhamnose but not xylose [9, 10]. However, our foodborne strains showed the absence of haemolysis after 24 hours of incubation on sheep blood agar contrary to the reference* L. monocytogenes* NCAIM B 01934 and presented it weakly only after 48 hours of incubation. The ISO standard refers to the possibility of the isolation of* L. monocytogenes* strains with weak haemolysis or negative CAMP test [10, 23]. Foodborne* L. monocytogenes* strains with either gene alteration or deletion of the* hly* gene or its regulatory protein PrfA are frequently detected, which cause weak haemolytic or nonhaemolytic phenotype in the atypical, aberrant, or nonhaemolytic strains [24, 25]. This phenomenon is highly probable for bacteria isolated from processed food or natural sources. The absence of haemolysis at 24 hours of incubation makes species identification hardly possible because only rhamnose utilization can be applied as a confirmation test by the standard method. At least four* Listeria* species (*L. monocytogenes*,* L. innocua*,* L. welshimeri*, and* L. grayi *ssp*. murray*) are known to utilize rhamnose and not xylose [10]. Therefore, a* Listeria* strain can be determined only at the genus level.

ALOA medium, which was inserted into the standard method [10], differentiates phospholipase positive* Listeria* colonies from another phosphatidylinositol negative* Listeria* [26]. On ALOA plates, all foodborne* Listeria *isolates produced blue-green colonies with an opaque halo. However, other strains than* L. monocytogenes* (e.g.,* L. seeligeri* and* L. ivanovii*) may also possess* plcA* gene coded phospholipase (PI-PLC) activity. Moreover, other bacteria species (e.g.,* Bacillus circulans* [27]) also possess phospholipase and *β*-glucosidase activities that resulted in halo and blue-green color of the colonies, respectively. Therefore, application of ALOA medium independently from the application of preliminary selective enrichment or other selective media (which also needs, at least, two days of incubation) could lead to false positive results.

Results of the biochemical analysis with the API* Listeria* test were compared with the reference profiles given by the manufacturer (bioMérieux, France). All of our foodborne* Listeria* isolates showed negative arylamidase (DIM), D-xylose (DXYL), and D-tagatose (TAG) tests (API* Listeria* numeric profile: 6510) (Table 1) and were suspected to be* L. monocytogenes*. It is generally regarded that the API test fails in some (10–15%) percent [23, 24, 28, 29]. The main reason of the misidentifications is hidden in the doubtfulness of the color determination of the weak arylamidase (DIM) test. The negative DIM test gives the basis for the differentiation of* L. monocytogenes *from the closely related* L. innocua* [30]. A weak positive DIM reaction can be evaluated as a negative result and can increase the number of the false positive determinations. Furthermore, false negative identification of atypical strains is also frequent [29, 31, 32].

A quite new technique for species identification is the Matrix Assisted Laser Desorption Time of Flight (MALDI-TOF) analysis. The isolates are differentiated on the basis of spectral peaks that typically correspond to highly abundant conserved proteins (e.g., ribosomal, cold shock, and nucleic acid binding proteins) that are so-called “housekeeping” proteins [33]. The results of the pattern matching process of the fingerprints obtained in MALDI-TOF MS analysis are expressed in score values (0–3.000). More than half of the samples (*n* = 18) were determined by 2.000–2.299 score values that mean secure identification only at genus level while only seven strains were given a highly probable species identification with higher than 2.300 scores. The identification revealed* L. monocytogenes*-*L. innocua* misidentification of the reference strains (*L. monocytogenes* ATCC 35152 and* L. innocua* ATCC 33090) with scores of high probability species determination of* L. innocua* in case of the* L. monocytogenes *ATCC 35152 atypical strain and for* L. seeligeri *ATCC 35967 gave* L. monocytogenes* or* L. innocua* (Table 1).

Our PCR primers were designed for conserved regions of the* hly* gene of LIPI-1 in* L. monocytogenes* and amplified a 438 bp fragment, while primer pair* hlyQF/hlyQR* was constructed for RT-PCR and amplified a 64 bp fragment from the* L. monocytogenes hly* gene [21]. Considering that only one band appeared with agarose gel electrophoresis, the tested* hlyQF/hlyQR* primer pair [21] was much reliable than, for example, that of Border et al. [34] with more amplificants. Interestingly, the* hly* gene detection with* hlyQF/hlyQR* primer pair revealed atypical strain determination for* L. seeligeri *ATCC 35967 (Table 1). It is well known that nonhaemolytic* L. seeligeri* possess a distinct LIPI-1 structure [4, 14]. Moreover, this primer pair also gave a reaction with atypical* L. monocytogenes* NCAIM B 01966. The specificity of the primer pair constructed in this work was higher than* hlyQF-hlyQR* or the primers of Aznar and Alarcón [29], because they received positive PCR results with their* hly* gene probes for other* Listeria* species (*L. ivanovii* and* L. seeligeri*). Furthermore, better than that of Cooray et al. [35], which failed to detect the* hly *gene from* L. monocytogenes* ATCC 15313 strain. With our primer pair, six out of eleven of the foodborne bacterial isolates gave positive reaction (Table 1), and we did not get a reaction with atypical or nonpathogenic* Listeria* strains.

## 4. Conclusions

Because of the close genetic relationship of the species and the usual presence of atypical strains in food, all tested species identification methods had drawbacks. The present work demonstrated that the standard tests [9, 10] are not always satisfactory for species determination and are not reliable for pathogenicity tests (e.g., haemolysis) of foodborne isolates.

MALDI-TOF MS is likely to become integral to microbiology laboratories for routine microorganism identification because of the short analysis time (<1 min per sample); thus one has the possibility to shorten the total identification time by at least 1-2 days. Here, some points should be addressed: (i) it needs a preliminary enrichment/purification of strains for the identification (needs at least 3 days) and (ii) the method needs validation because the settings of the software have great impact on the precision of the identification [36]. Additionally, identification of taxonomically closely related species has trouble, for example, differentiating* Listeria monocytogenes* from* Listeria innocua/Listeria ivanovii *[33]; therefore, it cannot be recommended for* Listeria* species differentiation in food quality control.

The PCR method is another possibility to shorten the identification time by detection of special genes without isolation steps. With our PCR primers, weak pathogenic or nonpathogenic* L. ivanovii*,* L. seeligeri*,* L. innocua,* and* L. welshimeri *strains and atypical nonhaemolytic* L. monocytogenes* were excluded.

It could be concluded that detection of potential pathogenicity is more important in food quality control than species determination as the specificity of the* Listeria monocytogenes* detection is 85.2% by the ISO standard method [10]. We propose that nucleic acid based detection of the LIPI-1 virulence gene cluster, which is a good marker of potential pathogenicity and does not need enrichment of the bacterium, should be used concomitantly with quick biochemical tests like phospholipase (PI-PLC) and *β*-glucosidase tests on selective ALOA medium to detect even atypical but pathogenic* Listeria* strains.

## Figures and Tables

**Table 1:** Summary of the test results of the reference and foodborne *Listeria* strains.

Bacteria tested	API test^a^	PCR with *hly *gene primers		MALDI-TOF MS score values^b^
		In this work	Rodríguez-Lázaro et al. [21]	
*L. monocytogenes* NCAIM B 01934 (ATCC® 15313*™*)	6510 *L. monocytogenes*	+	+	*L. monocytogenes* 2.430/2.312

*L. monocytogenes* NCAIM B 01966 (ATCC® 35152*™*)	6510	−	+	*L. innocua* 2.427/2.322/2.263/2.461

*L. innocua* NCAIM B01933 (ATCC® 33090*™*)	7510 *L. innocua*	−	−	*L. innocua* 2.151 *L. monocytogenes* 1.942

*L. welshimeri* NCAIM B01872 (ATCC® 35897*™*)	7311 *L. welshimeri*	−	−	*L. welshimeri* 2.211/2.109

*L. seeligeri* NCAIM B01873 (ATCC® 35967*™*)	3310 *L. seeligeri/ivanovii*	−	+	*L. monocytogenes * 2.241 *L. innocua* 2.179 *L. seeligeri* 2.050

*L. ivanovii* NCAIM B02035 (ATCC® 19119*™*)	3750 *L. ivanovii*	−	−	*L. ivanovii* 2.412/2.381

*L. grayi *ssp*. grayi* NCAIM B01871 (ATCC® 19120*™*)	7130 *L. grayi*	−	−	*L. grayi* 2.285/2.272

*L. grayi *ssp*. murray* NCAIM B01372 (ATCC® 25401*™*)	7130	−	−	*L. grayi* 2.266/2.283

Isolate 1	6510	−	+	*L. monocytogenes* 2.010/1.359

Isolate 2^c^	6510	−	+	*L. monocytogenes* 2.251/1.541

Isolate 3	6510	+	+	*L. monocytogenes* 2.118/2.083

Isolate 4	6510	+	+	*L. monocytogenes* 2.295/2.235

Isolate 5	6510	−	+	*L. monocytogenes* 2.245/2.229

Isolate 6	6510	+	+	*L. monocytogenes* 2.058/1.998

Isolate 7	6510	+	+	*L. monocytogenes* 2.314/2.349

Isolate 8	6510	+	+	*L. monocytogenes* 2.346/1.992

Isolate 9	6510	−	+	*L. monocytogenes* 2.246/2.045

Isolate 10	6510	+	+	*L. monocytogenes* 2.120/2.395

Isolate 11	6510	−	+	*L. monocytogenes* 2.380/2.258

^a^Numeric profiles.

^b^Results from repeated analyses.

^c^No growth on PALCAM agar.

+: detectable PCR product.

−: no PCR product.
